# Case of Calculous Concretion, &c.

**Published:** 1841-10

**Authors:** Willard Parker

**Affiliations:** Professor of Surgery and Surgical Anatomy in the College of Physicians and Surgeons of the University of the State of New York


*Case of Calculous Concretion forming around a fragment of a Bougie of the Slippery Elm.* By Willard Parker, M. D., Professor of Surgery and Surgical Anatomy in the College of Physicians and Surgeons of the University of the State of New York.

April, 1839. I was called to visit a patient at New Richmond, and requested to come prepared for the operation of lithotomy. The patient I found to be a man of naturally good constitution, a carpenter, married, and twenty-eight years old.

Upon inquiring into the history of the case it appeared, that three years before he had had some uneasiness in the urethra and difficulty in passing his urine. He applied to his family physician and soon obtained relief, and remained quite well until January, three months previous to my visit to him. He was then "down the river," and much exposed to wet and cold, after which his former difficulty in the urethra made its appearance again. He was advised to employ the slippery elm bougie, and proceeded at once to prepare one from the inner bark of the elm, fifteen inches long, and after immersing it in warm water for a few minutes, he succeeded in introducing it as directed, into the urethra; but when he attempted to withdraw the instrument it severed, and four inches were left behind. This almost immediately passed into the bladder,

Cystitis ensued at once, and the patient suffered intensely for a long time: at length, the severity of the symptoms abating, he was enabled to reach his residence. I found him greatly emaciated, pulse over one hundred, discharging from the bladder, as was judged by his attending physician, Dr.

Johnson, six or eight ounces of pus and mucus per diem. He had considerable suffering still about the neck of the bladder and along the urethra, and was unable to move without great distress. He had irritation at the rectum, tesnesmus, and some prolapsus.

There was no difficulty in the introduction of the sound: at the inferior fundus of the vesica urinaria was situated a large mass, immoveable, and having a gritty, yielding feel, as the sound was pressed upon it. On introducing the finger into the rectum, the foreign body could be distinctly felt, and it seemed impossible to remove it from its position.

It was decided that the mass must be removed, and for that purpose, the ordinary single lateral operation for stone was performed. After the incision into the bladder was made, and the forceps introduced, the bark could not be reached by them so as to be removed from its bed. The scoop was next employed, and by the aid of this instrument and the finger, the mass was loosened from its confinement and withdrawn, the bladder washed out with care, and the patient removed from the table to the bed.

Following the operation, there was considerable constitutional excitement for a few days, after the subsidence of which his health became better, the wound healed kindly, the discharge from the bladder soon ceased.

The points of interest in the above case, are, first, the nature of the nucleus, which was throughout encrusted with phosphatic deposit; and second, the fixedness of the mass at the bas-fond of the bladder. It is very probable that had the foreign substance been allowed to remain, ulceration would have taken place into the rectum, forming a recto-visical fistula, and from the irritation induced in the general system the patient would have succumbed.

The elm, it ought to be remarked, is a valuable article in surgery, as a tent to dilate fistulous openings, and were it not for its fragility it might be employed as a bougie for dilating strictures of the urethra. When the stricture is located just behind the glans penis, it may then be employed with safety. The elm, when used to dilate, must have been dried, and when shaped, then plunged into warm water for a few minutes, until its exterior becomes lubricated by the mucilage, and then it is introduced with much ease, and soon swells very much, and overcomes the constriction.—*N. Y. Med. Gaz.*

